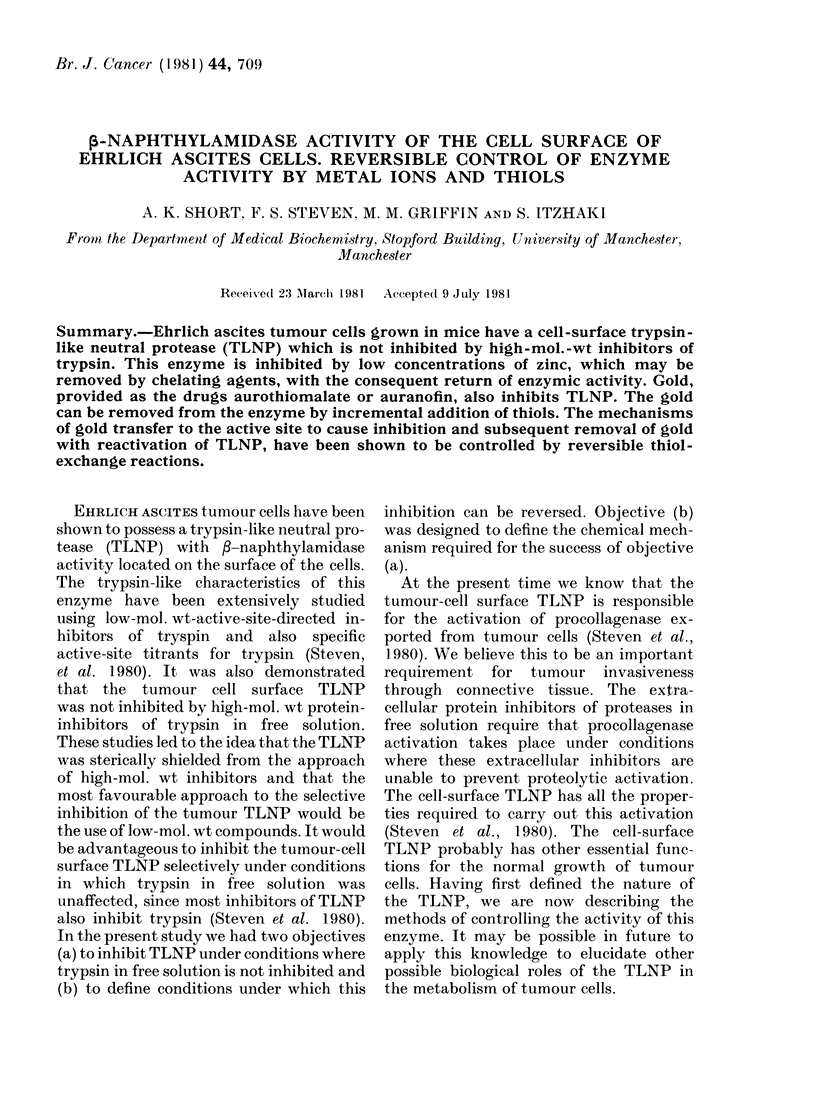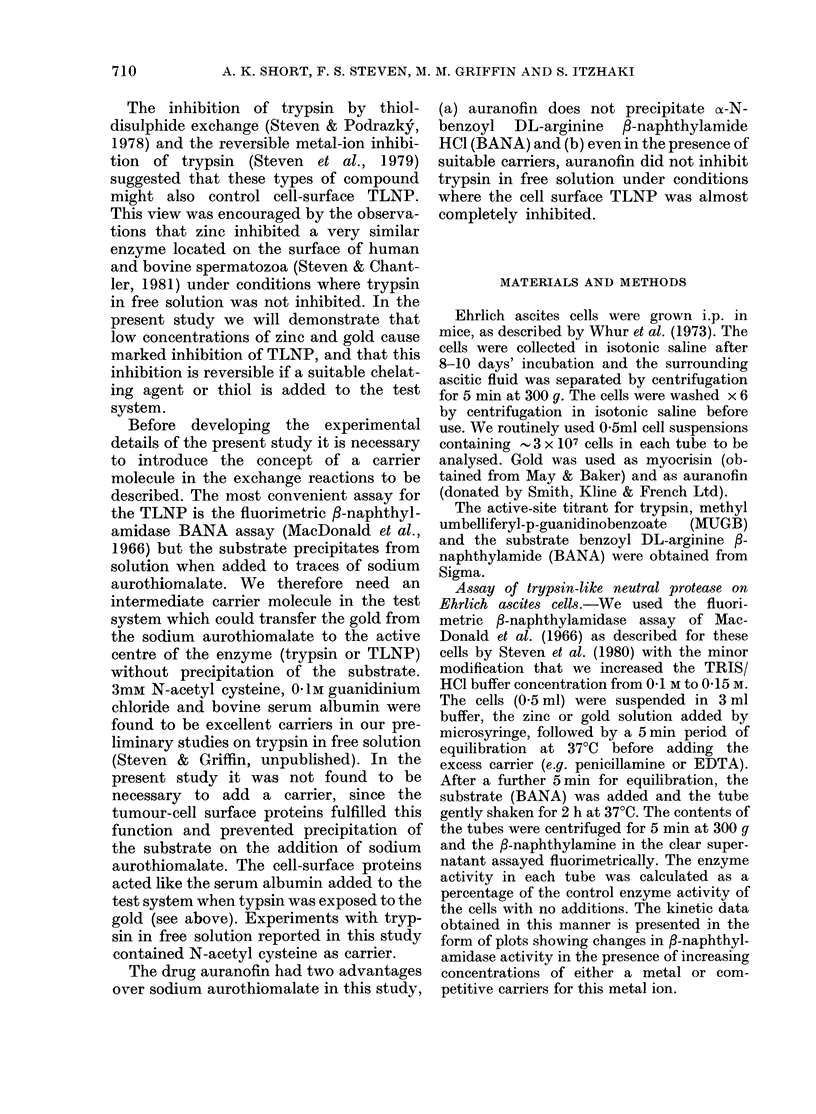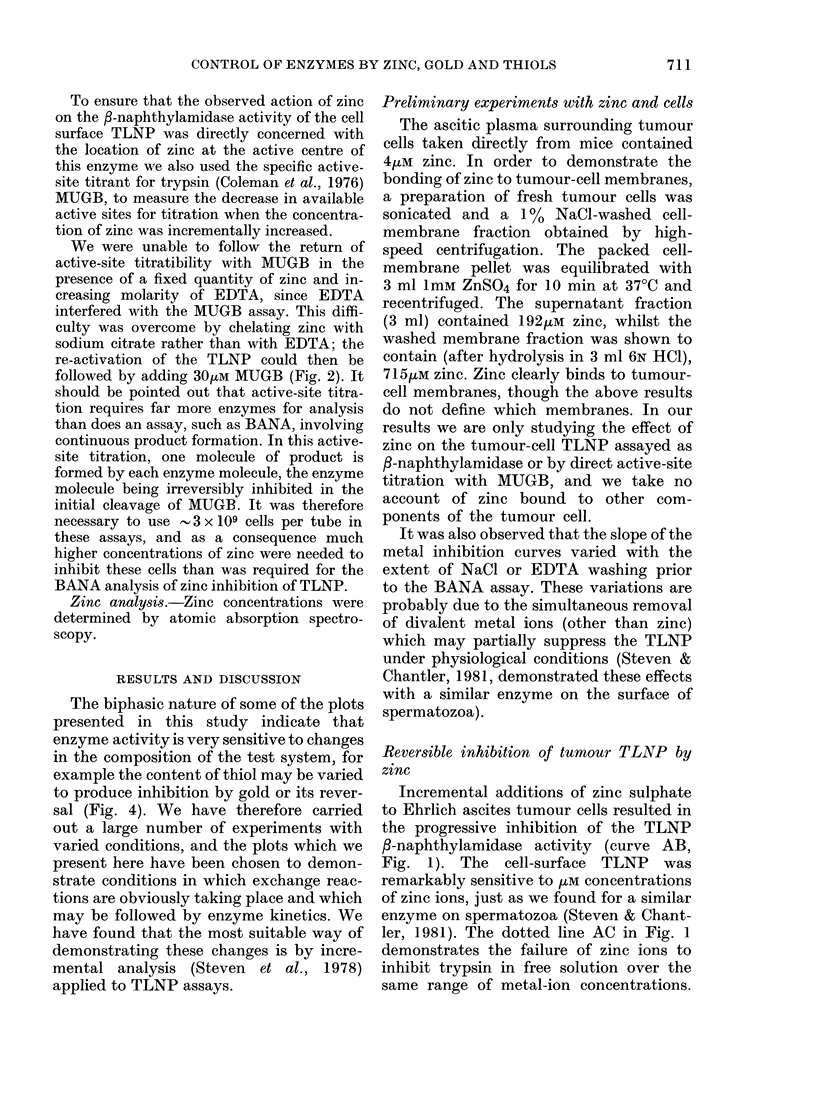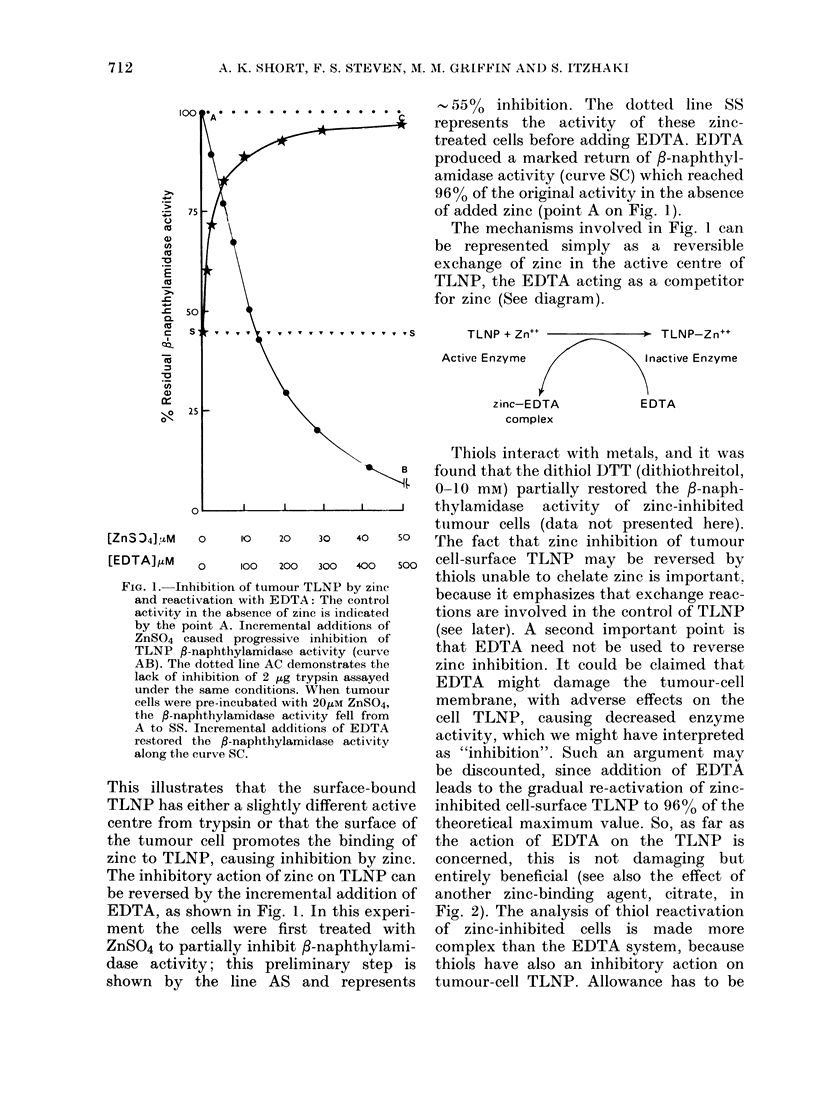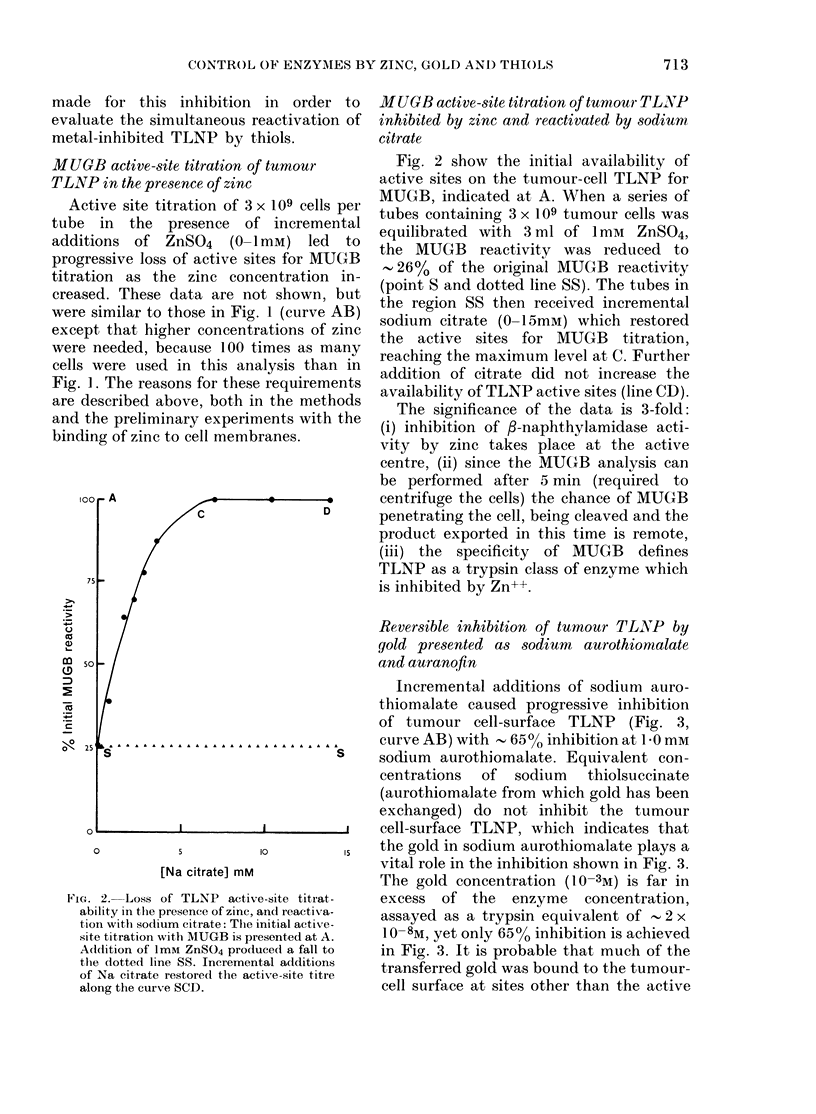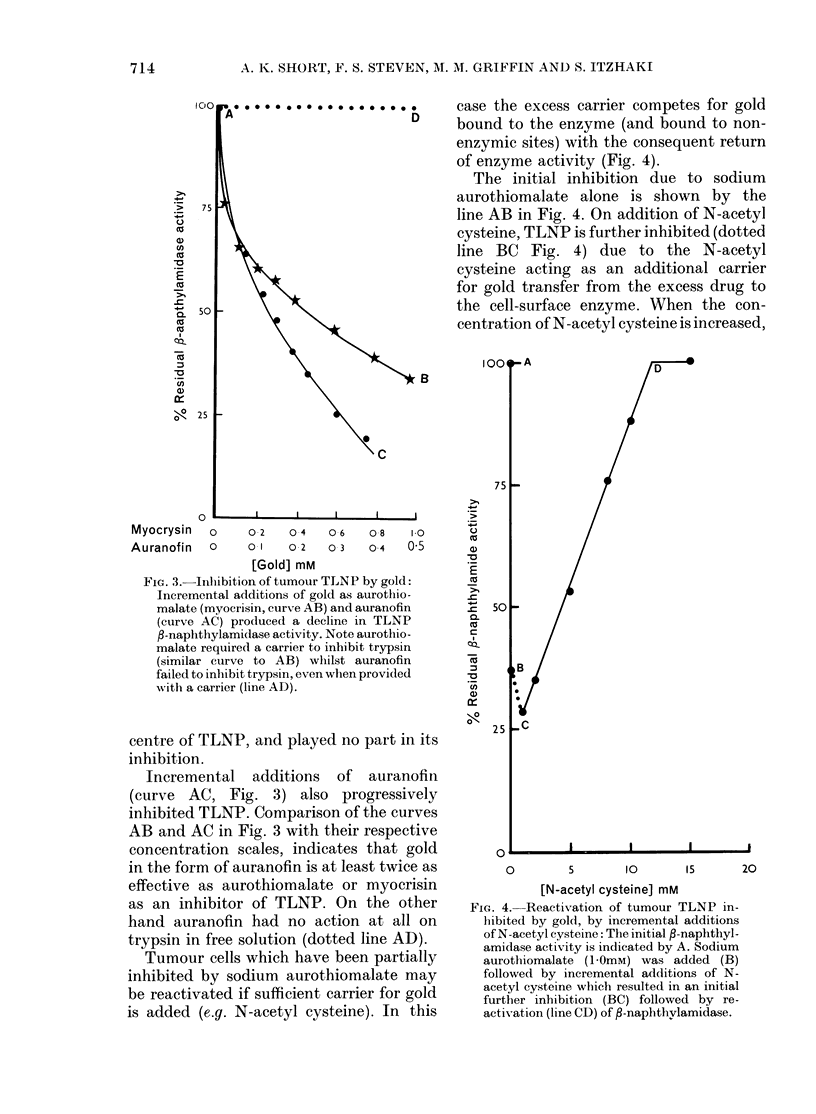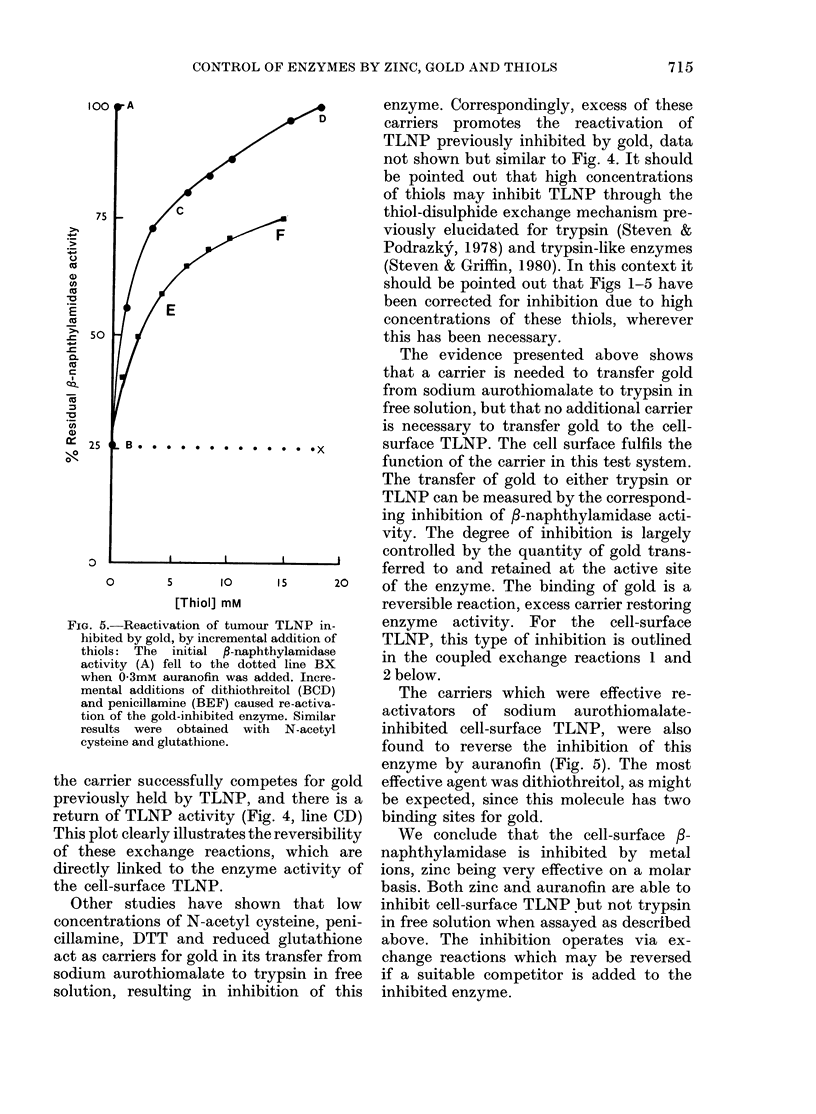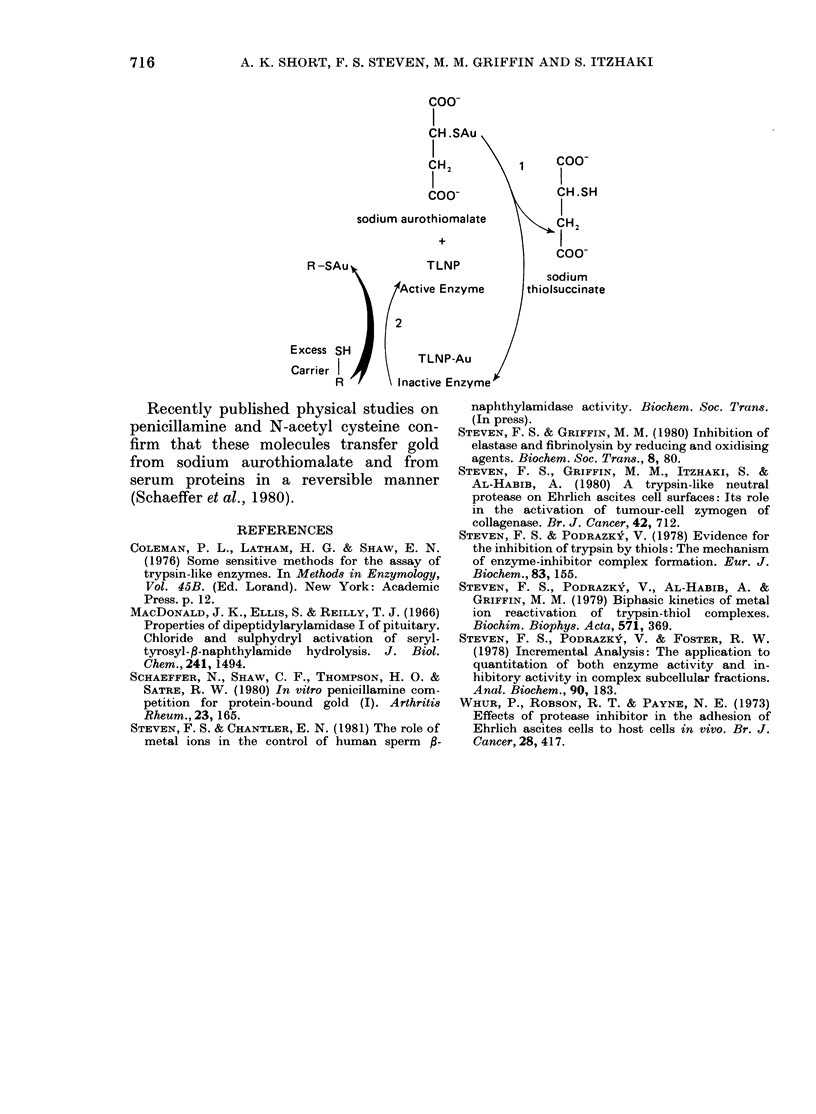# beta-Naphthylamidase activity of the cell surface of Ehrlich ascites cells. Reversible control of enzyme activity by metal ions and thiols.

**DOI:** 10.1038/bjc.1981.257

**Published:** 1981-11

**Authors:** A. K. Short, F. S. Steven, M. M. Griffin, S. Itzhaki

## Abstract

Ehrlich ascites tumour cells grown in mice have a cell-surface trypsin-like neutral protease (TLNP) which is not inhibited by high-mol.-wt inhibitors of trypsin. This enzyme is inhibited by low concentrations of zinc, which may be removed by chelating agents, with the consequent return of enzymic activity. Gold, provided as the drugs aurothiomalate or auranofin, also inhibits TLNP. The gold can be removed from the enzyme by incremental addition of thiols. The mechanisms of gold transfer to the active site to cause inhibition and subsequent removal of gold with reactivation of TLNP, have been shown to be controlled by reversible thiol-exchange reactions.


*Br. J. Cancer* (1981) **44**, 709

### β-NAPHTHYLAMIDASE ACTIVITY OF THE CELL SURFACE OF EHRLICH ASCITES CELLS. REVERSIBLE CONTROL OF ENZYME ACTIVITY BY METAL IONS AND THIOLS

A. K. SHORT, F. S. STEVEN, M. M. GRIFFIN AND S. ITZHAKI

*From the Department of Medical Biochemistry, Stopford Building, University of Manchester, Manchester*

Received 23 March 1981 Accepted 9 July 1981

**Summary.—Ehrlich ascites tumour cells grown in mice have a cell-surface trypsin-like neutral protease (TLNP) which is not inhibited by high-mol.-wt inhibitors of trypsin. This enzyme is inhibited by low concentrations of zinc, which may be removed by chelating agents, with the consequent return of enzymic activity. Gold, provided as the drugs aurothiomalate or auranofin, also inhibits TLNP. The gold can be removed from the enzyme by incremental addition of thiols. The mechanisms of gold transfer to the active site to cause inhibition and subsequent removal of gold with reactivation of TLNP, have been shown to be controlled by reversible thiol-exchange reactions.**

EHRLICH ASCITES tumour cells have been shown to possess a trypsin-like neutral protease (TLNP) with β-naphthylamidase activity located on the surface of the cells. The trypsin-like characteristics of this enzyme have been extensively studied using low-mol. wt-active-site-directed inhibitors of tryspin and also specific active-site titrants for trypsin (Steven, *et al.* 1980). It was also demonstrated that the tumour cell surface TLNP was not inhibited by high-mol. wt protein-inhibitors of trypsin in free solution. These studies led to the idea that the TLNP was sterically shielded from the approach of high-mol. wt inhibitors and that the most favourable approach to the selective inhibition of the tumour TLNP would be the use of low-mol. wt compounds. It would be advantageous to inhibit the tumour-cell surface TLNP selectively under conditions in which trypsin in free solution was unaffected, since most inhibitors of TLNP also inhibit trypsin (Steven *et al.* 1980). In the present study we had two objectives (a) to inhibit TLNP under conditions where trypsin in free solution is not inhibited and (b) to define conditions under which this inhibition can be reversed. Objective (b) was designed to define the chemical mechanism required for the success of objective (a).

At the present time we know that the tumour-cell surface TLNP is responsible for the activation of procollagenase exported from tumour cells (Steven *et al.*, 1980). We believe this to be an important requirement for tumour invasiveness through connective tissue. The extracellular protein inhibitors of proteases in free solution require that procollagenase activation takes place under conditions where these extracellular inhibitors are unable to prevent proteolytic activation. The cell-surface TLNP has all the properties required to carry out this activation (Steven *et al.*, 1980). The cell-surface TLNP probably has other essential functions for the normal growth of tumour cells. Having first defined the nature of the TLNP, we are now describing the methods of controlling the activity of this enzyme. It may be possible in future to apply this knowledge to elucidate other possible biological roles of the TLNP in the metabolism of tumour cells.

The inhibition of trypsin by thiol-disulphide exchange (Steven & Podrazký, 1978) and the reversible metal-ion inhibition of trypsin (Steven *et al.*, 1979) suggested that these types of compound might also control cell-surface TLNP. This view was encouraged by the observations that zinc inhibited a very similar enzyme located on the surface of human and bovine spermatozoa (Steven & Chantler, 1981) under conditions where trypsin in free solution was not inhibited. In the present study we will demonstrate that low concentrations of zinc and gold cause marked inhibition of TLNP, and that this inhibition is reversible if a suitable chelating agent or thiol is added to the test system.

Before developing the experimental details of the present study it is necessary to introduce the concept of a carrier molecule in the exchange reactions to be described. The most convenient assay for the TLNP is the fluorimetric $\beta$-naphthylamidase BANA assay (MacDonald *et al.*, 1966) but the substrate precipitates from solution when added to traces of sodium aurothiomalate. We therefore need an intermediate carrier molecule in the test system which could transfer the gold from the sodium aurothiomalate to the active centre of the enzyme (trypsin or TLNP) without precipitation of the substrate. 3mM N-acetyl cysteine, 0·1M guanidinium chloride and bovine serum albumin were found to be excellent carriers in our preliminary studies on trypsin in free solution (Steven & Griffin, unpublished). In the present study it was not found to be necessary to add a carrier, since the tumour-cell surface proteins fulfilled this function and prevented precipitation of the substrate on the addition of sodium aurothiomalate. The cell-surface proteins acted like the serum albumin added to the test system when typsin was exposed to the gold (see above). Experiments with trypsin in free solution reported in this study contained N-acetyl cysteine as carrier.

The drug auranofin had two advantages over sodium aurothiomalate in this study, (a) auranofin does not precipitate $\alpha$-N-benzoyl DL-arginine $\beta$-naphthylamide HCl (BANA) and (b) even in the presence of suitable carriers, auranofin did not inhibit trypsin in free solution under conditions where the cell surface TLNP was almost completely inhibited.

### MATERIALS AND METHODS

Ehrlich ascites cells were grown i.p. in mice, as described by Whur *et al.* (1973). The cells were collected in isotonic saline after 8–10 days' incubation and the surrounding ascitic fluid was separated by centrifugation for 5 min at 300 *g*. The cells were washed $\times 6$ by centrifugation in isotonic saline before use. We routinely used 0·5ml cell suspensions containing $\sim 3 \times 10^7$ cells in each tube to be analysed. Gold was used as myocrisin (obtained from May & Baker) and as auranofin (donated by Smith, Kline & French Ltd).

The active-site titrant for trypsin, methyl umbelliferyl-p-guanidinobenzoate (MUGB) and the substrate benzoyl DL-arginine $\beta$-naphthylamide (BANA) were obtained from Sigma.

*Assay of trypsin-like neutral protease on Ehrlich ascites cells.*—We used the fluorimetric $\beta$-naphthylamidase assay of MacDonald *et al.* (1966) as described for these cells by Steven *et al.* (1980) with the minor modification that we increased the TRIS/HCl buffer concentration from 0·1 M to 0·15 M. The cells (0·5 ml) were suspended in 3 ml buffer, the zinc or gold solution added by microsyringe, followed by a 5 min period of equilibration at 37°C before adding the excess carrier (*e.g.* penicillamine or EDTA). After a further 5 min for equilibration, the substrate (BANA) was added and the tube gently shaken for 2 h at 37°C. The contents of the tubes were centrifuged for 5 min at 300 *g* and the $\beta$-naphthylamine in the clear supernatant assayed fluorimetrically. The enzyme activity in each tube was calculated as a percentage of the control enzyme activity of the cells with no additions. The kinetic data obtained in this manner is presented in the form of plots showing changes in $\beta$-naphthylamidase activity in the presence of increasing concentrations of either a metal or competitive carriers for this metal ion.

To ensure that the observed action of zinc on the β-naphthylamidase activity of the cell surface TLNP was directly concerned with the location of zinc at the active centre of this enzyme we also used the specific active-site titrant for trypsin (Coleman *et al.*, 1976) MUGB, to measure the decrease in available active sites for titration when the concentration of zinc was incrementally increased.

We were unable to follow the return of active-site titratibility with MUGB in the presence of a fixed quantity of zinc and increasing molarity of EDTA, since EDTA interfered with the MUGB assay. This difficulty was overcome by chelating zinc with sodium citrate rather than with EDTA; the re-activation of the TLNP could then be followed by adding 30μM MUGB (Fig. 2). It should be pointed out that active-site titration requires far more enzymes for analysis than does an assay, such as BANA, involving continuous product formation. In this active-site titration, one molecule of product is formed by each enzyme molecule, the enzyme molecule being irreversibly inhibited in the initial cleavage of MUGB. It was therefore necessary to use $\sim 3 \times 10^9$ cells per tube in these assays, and as a consequence much higher concentrations of zinc were needed to inhibit these cells than was required for the BANA analysis of zinc inhibition of TLNP.

*Zinc analysis.*—Zinc concentrations were determined by atomic absorption spectroscopy.

### RESULTS AND DISCUSSION

The biphasic nature of some of the plots presented in this study indicate that enzyme activity is very sensitive to changes in the composition of the test system, for example the content of thiol may be varied to produce inhibition by gold or its reversal (Fig. 4). We have therefore carried out a large number of experiments with varied conditions, and the plots which we present here have been chosen to demonstrate conditions in which exchange reactions are obviously taking place and which may be followed by enzyme kinetics. We have found that the most suitable way of demonstrating these changes is by incremental analysis (Steven *et al.*, 1978) applied to TLNP assays.

### *Preliminary experiments with zinc and cells*

The ascitic plasma surrounding tumour cells taken directly from mice contained 4μM zinc. In order to demonstrate the bonding of zinc to tumour-cell membranes, a preparation of fresh tumour cells was sonicated and a 1% NaCl-washed cell-membrane fraction obtained by high-speed centrifugation. The packed cell-membrane pellet was equilibrated with 3 ml 1mM $ZnSO_4$ for 10 min at 37°C and recentrifuged. The supernatant fraction (3 ml) contained 192μM zinc, whilst the washed membrane fraction was shown to contain (after hydrolysis in 3 ml 6N HCl), 715μM zinc. Zinc clearly binds to tumour-cell membranes, though the above results do not define which membranes. In our results we are only studying the effect of zinc on the tumour-cell TLNP assayed as β-naphthylamidase or by direct active-site titration with MUGB, and we take no account of zinc bound to other components of the tumour cell.

It was also observed that the slope of the metal inhibition curves varied with the extent of NaCl or EDTA washing prior to the BANA assay. These variations are probably due to the simultaneous removal of divalent metal ions (other than zinc) which may partially suppress the TLNP under physiological conditions (Steven & Chantler, 1981, demonstrated these effects with a similar enzyme on the surface of spermatozoa).

### *Reversible inhibition of tumour TLNP by zinc*

Incremental additions of zinc sulphate to Ehrlich ascites tumour cells resulted in the progressive inhibition of the TLNP β-naphthylamidase activity (curve AB, Fig. 1). The cell-surface TLNP was remarkably sensitive to μM concentrations of zinc ions, just as we found for a similar enzyme on spermatozoa (Steven & Chantler, 1981). The dotted line AC in Fig. 1 demonstrates the failure of zinc ions to inhibit trypsin in free solution over the same range of metal-ion concentrations.

FIG. 1.—Inhibition of tumour TLNP by zinc and reactivation with EDTA: The control activity in the absence of zinc is indicated by the point A. Incremental additions of $ZnSO_4$ caused progressive inhibition of TLNP β-naphthylamidase activity (curve AB). The dotted line AC demonstrates the lack of inhibition of 2 μg trypsin assayed under the same conditions. When tumour cells were pre-incubated with 20μM $ZnSO_4$, the β-naphthylamidase activity fell from A to SS. Incremental additions of EDTA restored the β-naphthylamidase activity along the curve SC.

This illustrates that the surface-bound TLNP has either a slightly different active centre from trypsin or that the surface of the tumour cell promotes the binding of zinc to TLNP, causing inhibition by zinc. The inhibitory action of zinc on TLNP can be reversed by the incremental addition of EDTA, as shown in Fig. 1. In this experiment the cells were first treated with $ZnSO_4$ to partially inhibit β-naphthylamidase activity; this preliminary step is shown by the line AS and represents ~55% inhibition. The dotted line SS represents the activity of these zinc-treated cells before adding EDTA. EDTA produced a marked return of β-naphthylamidase activity (curve SC) which reached 96% of the original activity in the absence of added zinc (point A on Fig. 1).

The mechanisms involved in Fig. 1 can be represented simply as a reversible exchange of zinc in the active centre of TLNP, the EDTA acting as a competitor for zinc (See diagram).

Thiols interact with metals, and it was found that the dithiol DTT (dithiothreitol, 0–10 mM) partially restored the β-naphthylamidase activity of zinc-inhibited tumour cells (data not presented here). The fact that zinc inhibition of tumour cell-surface TLNP may be reversed by thiols unable to chelate zinc is important, because it emphasizes that exchange reactions are involved in the control of TLNP (see later). A second important point is that EDTA need not be used to reverse zinc inhibition. It could be claimed that EDTA might damage the tumour-cell membrane, with adverse effects on the cell TLNP, causing decreased enzyme activity, which we might have interpreted as "inhibition". Such an argument may be discounted, since addition of EDTA leads to the gradual re-activation of zinc-inhibited cell-surface TLNP to 96% of the theoretical maximum value. So, as far as the action of EDTA on the TLNP is concerned, this is not damaging but entirely beneficial (see also the effect of another zinc-binding agent, citrate, in Fig. 2). The analysis of thiol reactivation of zinc-inhibited cells is made more complex than the EDTA system, because thiols have also an inhibitory action on tumour-cell TLNP. Allowance has to be made for this inhibition in order to evaluate the simultaneous reactivation of metal-inhibited TLNP by thiols.

*MUGB active-site titration of tumour TLNP in the presence of zinc*

Active site titration of $3 \times 10^9$ cells per tube in the presence of incremental additions of $ZnSO_4$ (0–1mM) led to progressive loss of active sites for MUGB titration as the zinc concentration increased. These data are not shown, but were similar to those in Fig. 1 (curve AB) except that higher concentrations of zinc were needed, because 100 times as many cells were used in this analysis than in Fig. 1. The reasons for these requirements are described above, both in the methods and the preliminary experiments with the binding of zinc to cell membranes.

FIG. 2.—Loss of TLNP active-site titratability in the presence of zinc, and reactivation with sodium citrate: The initial active-site titration with MUGB is presented at A. Addition of 1mM $ZnSO_4$ produced a fall to the dotted line SS. Incremental additions of Na citrate restored the active-site titre along the curve SCD.

*MUGB active-site titration of tumour TLNP inhibited by zinc and reactivated by sodium citrate*

Fig. 2 show the initial availability of active sites on the tumour-cell TLNP for MUGB, indicated at A. When a series of tubes containing $3 \times 10^9$ tumour cells was equilibrated with 3 ml of 1mM $ZnSO_4$, the MUGB reactivity was reduced to ~26% of the original MUGB reactivity (point S and dotted line SS). The tubes in the region SS then received incremental sodium citrate (0–15mM) which restored the active sites for MUGB titration, reaching the maximum level at C. Further addition of citrate did not increase the availability of TLNP active sites (line CD).

The significance of the data is 3-fold: (i) inhibition of $\beta$-naphthylamidase activity by zinc takes place at the active centre, (ii) since the MUGB analysis can be performed after 5 min (required to centrifuge the cells) the chance of MUGB penetrating the cell, being cleaved and the product exported in this time is remote, (iii) the specificity of MUGB defines TLNP as a trypsin class of enzyme which is inhibited by $Zn^{++}$.

*Reversible inhibition of tumour TLNP by gold presented as sodium aurothiomalate and auranofin*

Incremental additions of sodium aurothiomalate caused progressive inhibition of tumour cell-surface TLNP (Fig. 3, curve AB) with ~ 65% inhibition at 1·0 mM sodium aurothiomalate. Equivalent concentrations of sodium thiolsuccinate (aurothiomalate from which gold has been exchanged) do not inhibit the tumour cell-surface TLNP, which indicates that the gold in sodium aurothiomalate plays a vital role in the inhibition shown in Fig. 3. The gold concentration ($10^{-3}$M) is far in excess of the enzyme concentration, assayed as a trypsin equivalent of $\sim 2 \times 10^{-8}$M, yet only 65% inhibition is achieved in Fig. 3. It is probable that much of the transferred gold was bound to the tumour-cell surface at sites other than the active

Fig. 3.—Inhibition of tumour TLNP by gold: Incremental additions of gold as aurothiomalate (myocrisin, curve AB) and auranofin (curve AC) produced a decline in TLNP β-naphthylamidase activity. Note aurothiomalate required a carrier to inhibit trypsin (similar curve to AB) whilst auranofin failed to inhibit trypsin, even when provided with a carrier (line AD).

centre of TLNP, and played no part in its inhibition.

Incremental additions of auranofin (curve AC, Fig. 3) also progressively inhibited TLNP. Comparison of the curves AB and AC in Fig. 3 with their respective concentration scales, indicates that gold in the form of auranofin is at least twice as effective as aurothiomalate or myocrisin as an inhibitor of TLNP. On the other hand auranofin had no action at all on trypsin in free solution (dotted line AD).

Tumour cells which have been partially inhibited by sodium aurothiomalate may be reactivated if sufficient carrier for gold is added (*e.g.* N-acetyl cysteine). In this case the excess carrier competes for gold bound to the enzyme (and bound to non-enzymic sites) with the consequent return of enzyme activity (Fig. 4).

The initial inhibition due to sodium aurothiomalate alone is shown by the line AB in Fig. 4. On addition of N-acetyl cysteine, TLNP is further inhibited (dotted line BC Fig. 4) due to the N-acetyl cysteine acting as an additional carrier for gold transfer from the excess drug to the cell-surface enzyme. When the concentration of N-acetyl cysteine is increased,

Fig. 4.—Reactivation of tumour TLNP inhibited by gold, by incremental additions of N-acetyl cysteine: The initial β-naphthylamidase activity is indicated by A. Sodium aurothiomalate (1·0mM) was added (B) followed by incremental additions of N-acetyl cysteine which resulted in an initial further inhibition (BC) followed by reactivation (line CD) of β-naphthylamidase.

FIG. 5.—Reactivation of tumour TLNP inhibited by gold, by incremental addition of thiols: The initial β-naphthylamidase activity (A) fell to the dotted line BX when 0·3mM auranofin was added. Incremental additions of dithiothreitol (BCD) and penicillamine (BEF) caused re-activation of the gold-inhibited enzyme. Similar results were obtained with N-acetyl cysteine and glutathione.

the carrier successfully competes for gold previously held by TLNP, and there is a return of TLNP activity (Fig. 4, line CD) This plot clearly illustrates the reversibility of these exchange reactions, which are directly linked to the enzyme activity of the cell-surface TLNP.

Other studies have shown that low concentrations of N-acetyl cysteine, penicillamine, DTT and reduced glutathione act as carriers for gold in its transfer from sodium aurothiomalate to trypsin in free solution, resulting in inhibition of this enzyme. Correspondingly, excess of these carriers promotes the reactivation of TLNP previously inhibited by gold, data not shown but similar to Fig. 4. It should be pointed out that high concentrations of thiols may inhibit TLNP through the thiol-disulphide exchange mechanism previously elucidated for trypsin (Steven & Podrazký, 1978) and trypsin-like enzymes (Steven & Griffin, 1980). In this context it should be pointed out that Figs 1–5 have been corrected for inhibition due to high concentrations of these thiols, wherever this has been necessary.

The evidence presented above shows that a carrier is needed to transfer gold from sodium aurothiomalate to trypsin in free solution, but that no additional carrier is necessary to transfer gold to the cell-surface TLNP. The cell surface fulfils the function of the carrier in this test system. The transfer of gold to either trypsin or TLNP can be measured by the corresponding inhibition of β-naphthylamidase activity. The degree of inhibition is largely controlled by the quantity of gold transferred to and retained at the active site of the enzyme. The binding of gold is a reversible reaction, excess carrier restoring enzyme activity. For the cell-surface TLNP, this type of inhibition is outlined in the coupled exchange reactions 1 and 2 below.

The carriers which were effective reactivators of sodium aurothiomalate-inhibited cell-surface TLNP, were also found to reverse the inhibition of this enzyme by auranofin (Fig. 5). The most effective agent was dithiothreitol, as might be expected, since this molecule has two binding sites for gold.

We conclude that the cell-surface β-naphthylamidase is inhibited by metal ions, zinc being very effective on a molar basis. Both zinc and auranofin are able to inhibit cell-surface TLNP but not trypsin in free solution when assayed as described above. The inhibition operates via exchange reactions which may be reversed if a suitable competitor is added to the inhibited enzyme.

Recently published physical studies on penicillamine and N-acetyl cysteine confirm that these molecules transfer gold from sodium aurothiomalate and from serum proteins in a reversible manner (Schaeffer *et al.*, 1980).

REFERENCES

COLEMAN, P. L., LATHAM, H. G. & SHAW, E. N. (1976) Some sensitive methods for the assay of trypsin-like enzymes. In *Methods in Enzymology, Vol. 45B*. (Ed. Lorand). New York: Academic Press. p. 12.

MACDONALD, J. K., ELLIS, S. & REILLY, T. J. (1966) Properties of dipeptidylarylamidase I of pituitary. Chloride and sulphydryl activation of seryl-tyrosyl-β-naphthylamide hydrolysis. *J. Biol. Chem.*, **241**, 1494.

SCHAEFFER, N., SHAW, C. F., THOMPSON, H. O. & SATRE, R. W. (1980) *In vitro* penicillamine competition for protein-bound gold (I). *Arthritis Rheum.*, **23**, 165.

STEVEN, F. S. & CHANTLER, E. N. (1981) The role of metal ions in the control of human sperm β-naphthylamidase activity. *Biochem. Soc. Trans.* (In press).

STEVEN, F. S. & GRIFFIN, M. M. (1980) Inhibition of elastase and fibrinolysin by reducing and oxidising agents. *Biochem. Soc. Trans.*, **8**, 80.

STEVEN, F. S., GRIFFIN, M. M., ITZHAKI, S. & AL-HABIB, A. (1980) A trypsin-like neutral protease on Ehrlich ascites cell surfaces: Its role in the activation of tumour-cell zymogen of collagenase. *Br. J. Cancer*, **42**, 712.

STEVEN, F. S. & PODRAZKÝ, V. (1978) Evidence for the inhibition of trypsin by thiols: The mechanism of enzyme-inhibitor complex formation. *Eur. J. Biochem.*, **83**, 155.

STEVEN, F. S., PODRAZKÝ, V., AL-HABIB, A. & GRIFFIN, M. M. (1979) Biphasic kinetics of metal ion reactivation of trypsin-thiol complexes. *Biochim. Biophys. Acta*, **571**, 369.

STEVEN, F. S., PODRAZKÝ, V. & FOSTER, R. W. (1978) Incremental Analysis: The application to quantitation of both enzyme activity and inhibitory activity in complex subcellular fractions. *Anal. Biochem.*, **90**, 183.

WHUR, P., ROBSON, R. T. & PAYNE, N. E. (1973) Effects of protease inhibitor in the adhesion of Ehrlich ascites cells to host cells *in vivo*. *Br. J. Cancer*, **28**, 417.